# Anthropogenic contamination of tap water, beer, and sea salt

**DOI:** 10.1371/journal.pone.0194970

**Published:** 2018-04-11

**Authors:** Mary Kosuth, Sherri A. Mason, Elizabeth V. Wattenberg

**Affiliations:** 1 University of Minnesota, School of Public Health, Division of Environmental Health Sciences, Minneapolis, Minnesota, United States of America; 2 State University of New York at Fredonia, Department of Chemistry and Biochemistry, Fredonia, New York, United States of America; Purdue University, UNITED STATES

## Abstract

Plastic pollution has been well documented in natural environments, including the open waters and sediments within lakes and rivers, the open ocean and even the air, but less attention has been paid to synthetic polymers in human consumables. Since multiple toxicity studies indicate risks to human health when plastic particles are ingested, more needs to be known about the presence and abundance of anthropogenic particles in human foods and beverages. This study investigates the presence of anthropogenic particles in 159 samples of globally sourced tap water, 12 brands of Laurentian Great Lakes beer, and 12 brands of commercial sea salt. Of the tap water samples analyzed, 81% were found to contain anthropogenic particles. The majority of these particles were fibers (98.3%) between 0.1–5 mm in length. The range was 0 to 61 particles/L, with an overall mean of 5.45 particles/L. Anthropogenic debris was found in each brand of beer and salt. Of the extracted particles, over 99% were fibers. After adjusting for particles found in lab blanks for both salt and beer, the average number of particles found in beer was 4.05 particles/L with a range of 0 to 14.3 particles/L and the average number of particles found in each brand of salt was 212 particles/kg with a range of 46.7 to 806 particles/kg. Based on consumer guidelines, our results indicate the average person ingests over 5,800 particles of synthetic debris from these three sources annually, with the largest contribution coming from tap water (88%).

## Introduction

The first peer-reviewed papers to document plastic pollution in the natural world were published over 45 years ago [1,2]. Since then, a robust body of work has accumulated, and the ubiquity of synthetic polymers in the environment is now undisputed. From abandoned gillnets hundreds of meters in length to plankton sized fragments, synthetic polymers have been extracted from remote corners of the Earth’s biosphere. Plastics have been quantified in marine environments [3] that include segments of the pelagic biome [4] coastal habitats [5], deep sea sediments [6, 7], as well as freshwater lakes [8,9] and associated tributaries [10]. Particles have also turned up in Arctic sea ice [11], ambient air [12], and a plethora of biota such as seabirds [13, 14], aquatic mammals [15], fish [16], and benthic invertebrates [17].

The last 45 years have also seen a commensurate increase in plastic production as the total global output of 30 million tons in 1970 climbed to 322 million tons in 2015 [18]. Hopes of closing the loop on the plastic waste stream depend on overall recycling rates, which vary widely across the globe, even among developed nations with well-established recycling infrastructure. Europe, for example, recycled 26% of disposable plastics in 2012, while the United States (US) reported rates as low as 8.8% in the same year [19].

The heterogeneous nature of microplastics make them a challenge to study. Although they are referred to in the literature as synthetic polymers derived from petrochemicals that are less than 5 mm in length, a universally accepted definition does not exist. Plastics in general represent a wide range of materials, each with unique physical characteristics and chemical compositions. Roughly 90% of plastic produced globally, however, falls into one of six categories: HDPE, LDPE, PP, PVC, PS, and PET [20].

Plastics are hydrophobic and have been known to adsorb chemicals from the environment such as PCBs, PBDEs, and PAHs [21], some of which are known reproductive toxicants and carcinogens [22, 23, 24]. Plastic can also adsorb metals [25] and bacteria [26], sometimes at concentrations many times higher than their immediate surroundings [27]. Furthermore, there is evidence that once ingested some of these organic chemicals can desorb in the guts of animals [28]. Plastics can also leach synthetic additives, such as phthalates, alkylphenols, and bisphenol A [29]. A more recent study indicates that plastics can be cytotoxic to human cells [30]. Finally, plastic debris can serve as a unique microhabitat for marine organisms [31, 32] and aid in the transport of invasive species [33]. These known issues highlight why microplastics are considered a contaminant of emerging concern [34, 35, 36].

While evidence of plastic pollution in the natural world quickly mounts, few studies focus on synthetic polymer contamination in human consumables. A 2014 publication reported synthetic polymers in 24 brands of German beer [37]. Another study published the following year found microplastics in 15 brands of Chinese commercial salt sourced from lakes, mines, and coastal seas [38]. Two more studies of salt emerged in 2017; one reported the presence of plastic particles in globally sourced commercial salt [39] while the other found plastic particles in Spanish table salt [40]. Anthropogenic debris was also found in both fish and bivalves that were purchased in markets, intended for human consumption [35]. The known accumulation of anthropogenic debris in global water bodies makes contamination of human consumables sourced from those water bodies very likely. This study and others that predate it, seek to provide evidence of this contamination.

Our study focused on three common human consumables: beer, sea salt, and tap water. One objective of this study was to determine if the findings from previous studies [32, 33, 34, 35] regarding beer and salt are regional anomalies or pieces of a larger, global food and beverage contamination issue. For this reason, we analyzed contamination of beer and salt products purchased within the US [33, 34, 35]. We specifically analyzed beers brewed from water sourced from the Laurentian Great Lakes because of the known prominence of plastic pollution within those bodies of water. Internationally sourced salts purchased in the city of Minneapolis were chosen because when it comes to products such as salt, local markets often sell globally sourced products.

Another major objective of this study was to begin surveying contamination of drinking water. To the authors’ knowledge, no survey of anthropogenic debris in tap water has ever been published. We analyzed 159 water samples collected from fourteen countries. The samples, provided by Orb Media, span seven geographical regions from five continents. Approximately half of the samples came from developed countries and the other half from developing countries. The samples, representing both rural and urban communities, were subjected to different filtering methods and were used for different purposes. This broad survey provides an indication of whether the levels of contamination differ between developing and developed nations, and serves as a foundation for future studies that can focus on more specific questions regarding tap water contamination.

## Materials and methods

### Sample collection

#### Tap water

Tap water samples (n = 159 total; Table 1) were collected between January and April of 2017 from the following 14 countries: Cuba (n = 1), Ecuador (n = 24), England (n = 3), France (n = 1), Germany (n = 2), India (n = 17), Indonesia (n = 21), Ireland (n = 1), Italy (n = 1), Lebanon (n = 16), Slovakia (n = 8), Switzerland (n = 2), Uganda (n = 26), and the US (n = 36). Some of these samples were collected by Orb Media’s institutional partners, three of which were professional scientific service organizations, namely Difaf in Beirut, Lebanon, EarthGreen in Quito, Ecuador, and ToxicsLink in Delhi, India and two of which were non-scientific partners, namely Jibu in Kampala, Uganda, and Klirkom in Jakarta, Indonesia. The rest of the samples were collected by Orb Media staff members and volunteers stationed around the world. Three of the US samples were obtained as bottled water.

**Table 1:** General information about the 159 tap water samples analyzed in this study.

Country	City(ies)	No. Samples	Filtered at residence?
Cuba	Havana	1	No
Ecuador	Quito	24	No
England	London	3	No
France	Paris	1	No
Germany	Berlin(1); Tubingen(1)	2	No
India	New Delhi	17	Yes(4); No(13)
Indonesia	Depok City (1); Desa Puspanegara(1); Jakarta(10); Jatirahayu (1); Kedaung(1); Menteng(1); North Paninggilan(1); Pasireurih(1); South Tangerang City(1); Sukatani(1); Teluknaga(1); Warnasari(1)	21	Yes(1); No(20)
Ireland	Dublin	1	No
Italy	Pavia	1	No
Lebanon	Beirut(11); Burj el Brajneh (1); Choueifat (1); Ghobayreh(1); Khaldeh(1); Mreijeh(1)	16	No
Slovakia	Brezová pod Bradlom(2); Kočovce(1); Piešťany(1); Poprad(1); Prašník(1); Radošina(1); Ružomberok(1)	8	No
Switzerland	Davos Platz(1); Gerbertingen(1)	2	No
Uganda	Jinja(1); Kampala(25)	26	Yes(2); No(24)
USA	Alpena(1); Buffalo(1); Chicago(3); Clayton(1); Duluth(1); Glenview(1); Holland(1); Lawrence (1); Los Angeles(3); Louisville(1); Mahopack(1); Middle Village(1); New York City(6); Overland Park(1); Palmetto Bay(1); Pinebluff(1); Spring(1); Washington DC(6); Wauwatosa(1)	33	Yes(6); No(26)
USA	Bottled Water	3	Yes

Most samples (n = 156; 98%) were collected by running the tap water source for 1 minute prior to filling a 500mL HDPE bottle to the point of overflowing. While leaving the water running, the bottle was filled twice and dumped twice before being filled a third time and capped. This was done to rinse the bottle prior to the final sample collection. A survey was filled out for each water sample, which included the sample collector’s name and contact information, day and time of collection, and information about the source and general use of the water taken (Table 1). The survey form was then mailed to Orb Media, who tracked the samples, while the water sample itself was sent to the University of Minnesota, Minneapolis for processing. Three of the 159 total samples (2%) were obtained as bottled water by Orb Media staff. These samples were poured directly from the water bottle into identical 500 mL HDPE bottles immediately after opening and mailed in a fashion identical to the other tap water samples. Because the water samples, identified only through a unique sample ID number, were tracked by one organization and processed by another organization they were processed ‘blind’ without any preconceptions about the tap water source.

#### Statistical analysis

The data for each country was expressed as a mean with standard deviation. We also compared the mean of all developed nations to the mean of all developing nations. Averages of developed countries were compared to developing countries using a Welch t-test.

#### Beer

Twelve brands of beer were purchased between January and April of 2017. All of the beer manufacturers used municipal water (representing a total of nine municipalities) drawn from one of the five Laurentian Great Lakes. Each brewery was reached by phone or email to confirm their water source. The water treatment facility for each of the nine municipalities was also reached by phone to confirm their water source. Three breweries drew water from Lake Superior, four from Lake Michigan, one from Lake Huron, two from Lake Erie, and two from Lake Ontario (Table 2). Seven brands of beer were purchased from Minneapolis, Minnesota liquor stores, two were purchased directly from breweries in Duluth, Minnesota, and the remaining three were purchased in Alpena, Michigan and Rochester, New York. All beers were packaged in 12- or 16-fluid-ounce aluminum cans, 12-fluid-ounce glass bottles, 64-fluid-ounce glass growlers, or 32-fluid-ounce glass howlers (Table 2).

**Table 2:** General information about the 12 beers analyzed for this study.

WATER SOURCE/ BEER ID	CONTAINER MATERIAL	OZ PRODUCT/CONTAINER	# OF LOTS
Lake Superior 1	Glass	2–64 fl oz growlers	1
Lake Superior 2	Glass	2–64 fl oz growlers	1
Lake Superior 3	Aluminum	9–12 oz cans	---
Lake Michigan 1	Glass	9–12 oz bottles	3
Lake Michigan 2	Glass	9–12 oz bottles	1
Lake Michigan 3	Aluminum	7–16 oz cans	3
Lake Michigan 4	Glass	9–12 oz bottles	3
Lake Huron 1	Aluminum	9–12 oz cans	6
Lake Erie 1	Glass	9–12 oz bottles	5
Lake Erie 2	Glass	9–12 oz bottles	2
Lake Ontario 1	Glass	3–32 fl oz howlers	1
Lake Ontario 2	Aluminum	7–16 oz cans	---

Tap water samples from seven of the nine municipalities represented within the 12 beer brands analyzed were obtained as part of the tap water study above, following the same sampling guidelines discussed. These samples were obtained specifically to investigate any possible correlation between the number of particles found in the tap water supply and those found in beer.

#### Sea salt

Twelve brands of sea salt were purchased in August of 2016 from six grocery stores and specialty shops in Minneapolis, Minnesota. Brands were selected based on their region of origin, which was detailed on each product’s label (Table 3). Effort was made to select brands sourced from different regions of the world. Ten brands were sourced from oceans (n = 4) and seas (n = 6), while two came from salt mines. Six salt brands were packaged in plastic bags, two were packaged in stiff cardboard cylinders, two in glass jars, and two in plastic containers (Table 3).

**Table 3:** General information about the 12 salts analyzed in this study.

SALT ID	SOURCE	CONTAINER SIZE	CONTAINER MATERIAL
North Sea Salt	Seas	240 g	1 plastic bag
Celtic Sea Salt 1	Seas	453 g	1 plastic bag
Celtic Sea Salt 2	Seas	227 g	1 plastic bag
Sicilian Sea Salt	Seas	102 g	2 glass jars
Mediterranean Sea Salt 1	Seas	750 g	1 plastic cylinder (PP)
Mediterranean Sea Salt 2	Seas	750 g	1 cardboard cylinder
Utah Sea Salt	Mined	283 g	1 plastic cylinder (PET)
Himalayan Rock Salt	Mined	500 g	1 glass jar
Hawaiian Sea Salt	Ocean	340 g	1 plastic bag
Baja Sea Salt	Ocean	454 g	1 plastic bag
Atlantic Sea Salt	Ocean	907 g	1 plastic "biodegradable" bag
Pacific Sea Salt	Ocean	737 g	1 cardboard cylinder

### Sample processing

#### Tap water

Tap water samples (n = 159) were processed for anthropogenic particles using a method similar to that used by Liebezeit et al. (2014) [15], briefly described below. Since the samples were collected by volunteers, the volumes of the samples varied (ranging from 457 to 603 ml, with a mean of 551 ml) and as such, volumes were recorded prior to vacuum filtration through a 55 mm diameter Whatman cellulose filter with a pore size of 2.5 μm. To ensure complete evacuation, sample bottles were rinsed three times with deionized water, with the rinse water being passed through the same filter as the original sample. After each water sample was filtered and rinsed in triplicate, the filtrate was itself passed through a second new filter and cleaned glassware. This second filtration was carried out to examine the possible ‘break through’ of contaminants. For example, given the small diameter of synthetic fibers, it is possible that they could pass through a filter even if their length was prohibitive. Any particles found in the second filtration were added to the particles found in the corresponding sample.

In order to aid the visualization of anthropogenic (synthetic) debris, 2 ml of the biological stain Rose Bengal, at a concentration of 200 mg/L, was applied to each filter with an eyedropper. Filters were visually analyzed using a dissection microscope (Leica EZ4W, 8-35X Zoom, Integrated 5MP Camera). Particles not stained by the Rose Bengal were agitated with a stainless steel micro spatula to test each particle’s durability. Each piece that was able to endure this test without breaking apart was identified as anthropogenic debris [15] and was measured, catalogued, and photographed at 35X. To determine fiber length, a metric ruler was used to demark measurements on the stainless steel spatula that was also used to test the resiliency of found fibers. Fiber length was enumerated based on measurement of the fiber to these demarcations. While fiber color is somewhat subjective, broad categories were used to limit subjectivity based upon the observer. All filters were stored in individual Petri dishes for possible future analysis.

It is important to note that while particles not stained by the Rose Bengal were classified and referred to as “microplastic” in Liebezeit et al. (2014) [15], we are choosing to use the more general term “anthropogenic debris.” Given that Rose Bengal is a biological stain and thus should bind to natural materials/fibers, it is logical to assume that the particles found are at least synthetic and most likely could be classified as microplastics, but as spectroscopic analyses such as fourier transform infrared spectroscopy (FTIR) are required in order to confirm this assumption, we use the more general term throughout this report.

Although the pore size of the filter used for tap water (2.5 μm) is smaller than the pore size of the filter used for beer and salt (11 μm) (see below), the mean number of particles were similar, the mean density was similar, and the fiber sizes were similar between beer and water, indicating that the difference in pore size did not limit the detection of particles. It is possible that the different pore size affected filtering time, but not in a significant way.

#### Beer

Beer samples were processed using a method similar to that used for tap water described above. Exactly 1 L of beer was measured and vacuum-filtered through a 70-mm-diameter Whatman cellulose filter with a pore size of 11 μm. Again, sample bottles and cans were rinsed three times with deionized water, which was passed through the same filter. Filters were treated with 6 ml of a 200 mg/L concentration of Rose Bengal, the same biological stain used in the tap water study, before they were visually analyzed under a dissection microscope. Particles that were able to endure the test of durability were measured using the same tools and methods described in the water study, catalogued, and photographed at 35X. All filters were stored in individual Petri dishes for possible future analysis.

Tap water samples collected for comparison to the beer samples (seven samples from the nine municipalities represented within the 12 beer brands) were processed in the same manner as the tap water samples above.

#### Sea salt

Sea salt samples were processed using a method similar to that used for the beer samples described above. Exactly 50 g of a salt was measured and dissolved in 1 L of millipore deionized water before it was vacuum-filtered through a 70-mm-diameter Whatman cellulose filter with a pore size of 11 μm. The volumetric flask was rinsed three times with deionized water, which was passed through the same filter. Filters were stained with 6 ml of Rose Bengal at a 200 mg/L concentration before they were visually analyzed with a dissection microscope. Particles that were able to endure the test of durability were measured, using the same tools and methods described in the water and beer study, catalogued, and photographed at 35X. All filters were stored in individual Petri dishes for possible future analysis.

### Quality assurance and quality control

In order to prevent/reduce potential contamination throughout the sample processing from external sources, such as airborne fibers, all glassware was covered with a watch glass when not in use and washed thoroughly between trials. Work occurred in a laminar airflow cabinet, and the workspace was wiped down every week. Filters were inspected under a microscope prior to use. Filtration times were recorded so that the window of time for potential contamination was known. Finally, a cotton lab coat and sterling nitrile powder free exam gloves were worn throughout the experimental procedure.

To further account for contamination, two different types of lab blanks were processed. For the tap water samples alone, bottle blanks were run by filling two empty 500 mL HDPE bottles with deionized water in the lab, just as the tap water samples themselves had been collected. Additionally, for all three studies (tap water, beer and sea salt) lab blanks containing only deionized water were run. These blanks were called deionized blanks and were carried out to account for background lab contamination from atmospheric deposition, deionized water, and glassware. For the tap water study, deionized blanks were run once each day that samples were processed (n = 30), while for the beer and salt studies one blank was processed for each brand (n = 12 for each; n = 24 total). The bottles and deionized blanks were processed in a manner identical to the samples themselves in order to account for possible anthropogenic contamination that could be coming from the either the collection receptacle or testing environment.

## Results

### Quality control: Laboratory blanks

**Fig 1:**
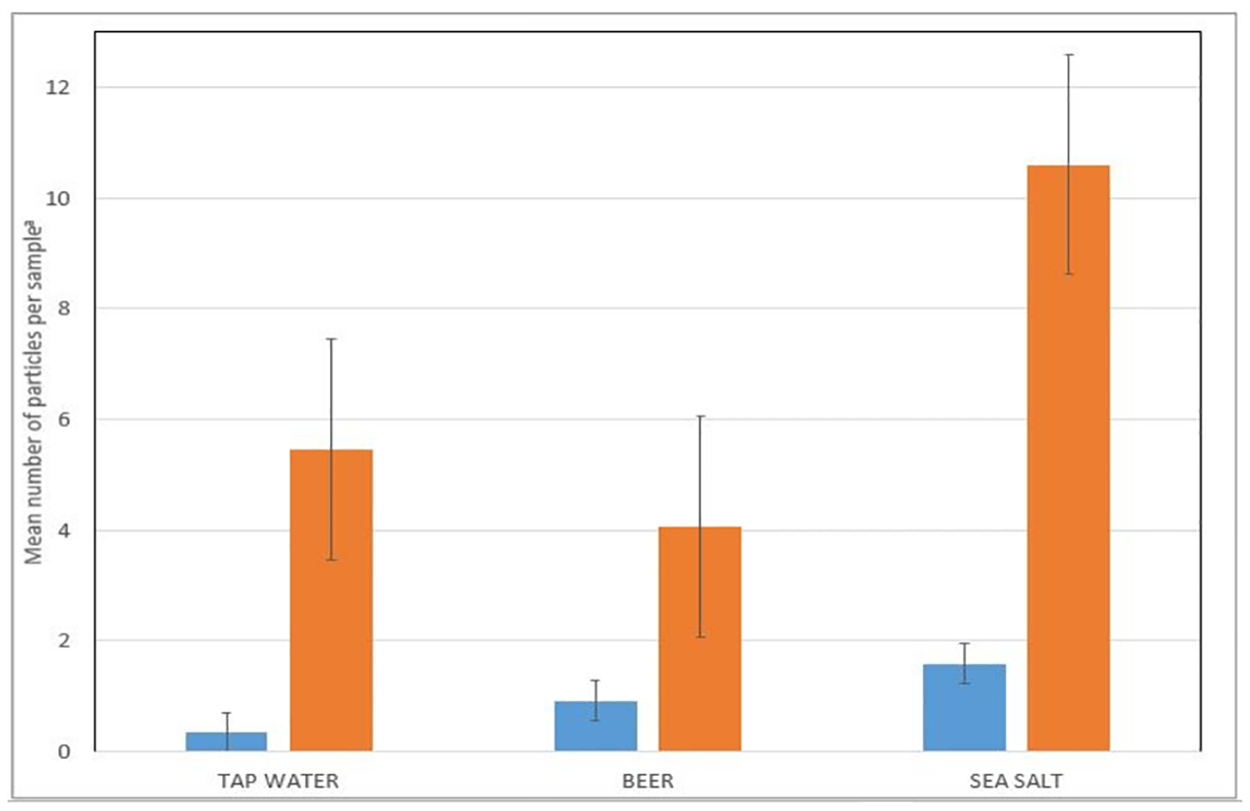
Summary of laboratory blanks. Averages of laboratory blanks processed as part of tap water (n = 30), beer (n = 12) and salt (n = 12) studies. Error bars indicate the standard deviation of the results.

Of the 30 total deionized blanks processed as part of the tap water study (one each day that water samples were processed) 5 had one anthropogenic particle in it and the others had none. For the beer study a total of 12 deionized blanks were run yielding 6 with one anthropogenic particle, 1 with two particles, 1 with three particles, and the remaining 4 containing no particles. Of the 12 deionized blanks run for each brand of salt, 5 had one anthropogenic particle, 1 had two particles, 1 had three particles, 1 had four particles, 1 had five particles, and the remaining 3 had no particles. Fig 1 illustrates the averages and standard deviations of these results in comparison to the samples themselves.

In summary, of the 54 total deionized water blanks processed over the course of the experiments, 11 contained one particle, 7 contained two particles, 2 contained three particles, 1 contained four particles, and 1 contained five particles, while the remaining 32 (60%) contained zero particles. In total the deionized blanks indicate that there was little to no background laboratory contamination within the samples processed. Nevertheless, in order to be conservative, counts obtained within lab blanks were subtracted from the total for each sample as explained in more detail below.

### Tap water

During the first four months of 2017, tap water samples were collected from 14 countries worldwide, representing seven distinct regions. Samples were processed individually with the number of anthropogenic particles per sample calculated as the sum of the number of particles within the first and second filtration less the number of particles found within that day’s deionized blank. Given some variability in sample volume, the density of anthropogenic particle contamination was calculated as the number of particles per liter of water (particle/L) in order to standardize the samples.

Anthropogenic debris was found in 81% of the 159 samples tested. The range of anthropogenic particles within all tap water samples was 0 to 61 particles/L, with an overall mean of 5.45 particles/L. The highest mean for any country was found in the US with 9.24 particles/L while the four lowest means were from European Union (EU) nations. (Table 4). Three brands of bottled water were also included in the study. The average for these non-municipal water sources was 3.57 particles/L, which was less than the overall average. Interestingly, when the mean of all developing countries was compared with the mean of all the developed countries, a statistically significant difference (p = 0.025) was found between the two groups. Water sourced from more developed nations (EU, US, and Lebanon) had an average density of 6.85 particles/L, while water sourced from less developed nations (Cuba, Ecuador, India, Indonesia, Uganda) had an average density of 4.26 particles/L.

**Table 4:** Summary of tap water results.

		Particles Per Liter^a^			
COUNTRY/SOURCE	NO. SAMPLES	MINIMUM	MAXMUM	MEAN	STD. DEV.
Cuba	1	---	---	7.17	---
Ecuador	24	0	9.04	4.02	3.20
England	3	3.66	13.0	7.73	4.76
France	1	---	---	1.82	---
Germany	2	0	1.82	0.91	1.29
India	17	0	20.0	6.24	6.41
Indonesia	21	0	10.8	3.23	3.48
Ireland	1	---	---	1.83	---
Italy	1	---	---	0^b^	---
Lebanon	16	0	23.3	6.64	6.38
Slovakia	8	0	10.9	3.83	4.47
Switzerland	2	0	5.47	2.74	3.87
Uganda	26	0	12.7	3.92	3.17
USA	33	0	60.9	9.24	11.8
Bottled Water	3	1.78	5.37	3.57	1.79

^a^For countries with only one sample, the density of anthropogenic debris is provided as the mean with no values given for min., max., or standard deviation.

^b^While anthropogenic debris was found within this sample, the sample itself had less than the deionized blank and, thus, its value is listed as zero.

**Fig 2:**
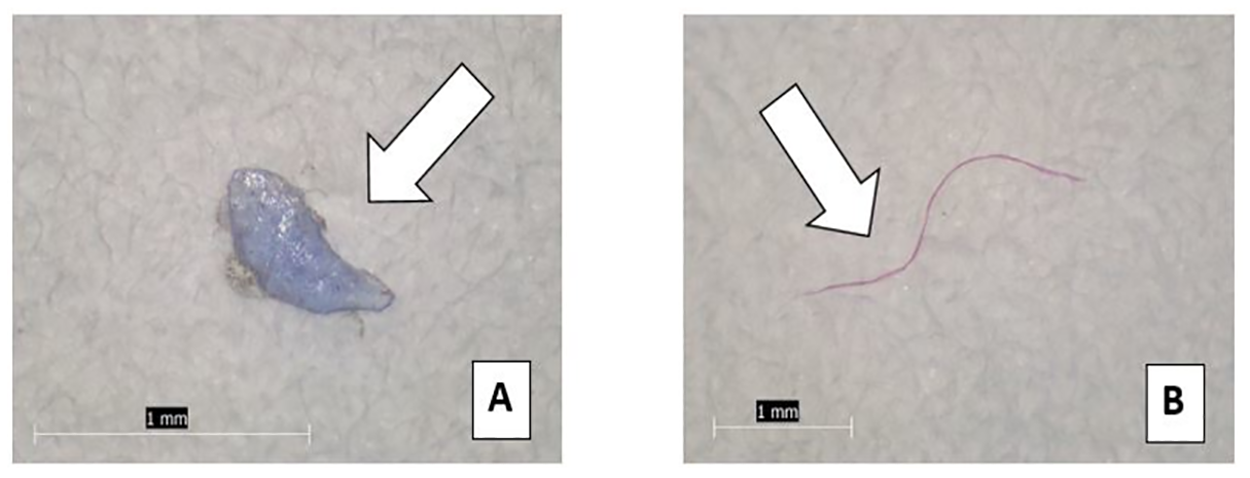
Tap water particles. Examples of anthropogenic particles found in tap water: (A) Fragment, 1 mm in length from Indian subcontinent; (B) Fiber, 2.5 mm in length from U.S. tap water sample.

**Fig 3:**
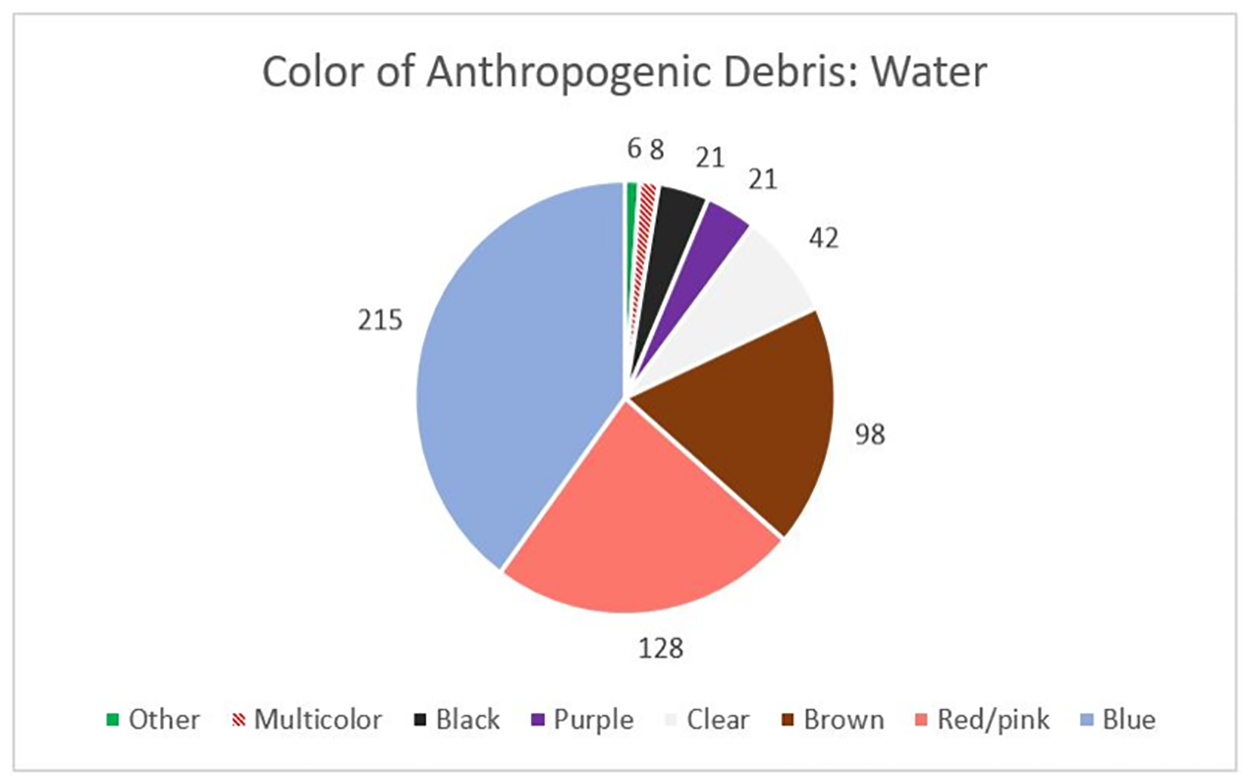
Tap water particle colors. Color distribution of anthropogenic particles extracted from 159 samples of tap water.

Of the 539 particles found, the vast majority (98.3%) were identified as fibers, and the remaining particles were identified as fragments (n = 7) or films (n = 2) (Fig 2). The fibers varied in length from 0.10–5.00 mm, with an average of 0.96 mm. Of the 159 water samples, 66 (41.5%) had one or more particles, specifically fibers, in the second filtration step, indicating the difficulty of complete fiber removal via filtration. These particles had an average length of 0.85 mm, which is 0.11 mm smaller than the particles found in the samples as a whole. Of the 539 particles found, the most common color was blue, followed by red/pink, and brown (Fig 3). We felt it important to take note of particle color as some studies have indicated that aquatic organisms may preferentially ingest certain colors of debris over others. It may also be useful in determining source of debris.

### Beer

Anthropogenic debris was found in all 12 brands of beer that were tested. For each brand, three separate, one-liter samples were processed. One of the three samples was selected at random to be filtered a second time. It was assumed that particles from the second filtration were breakthrough particles and therefore added to the sample total. The number of particles per liter was then calculated as the average of the three samples less the number of particles found in the corresponding deionized blank in order to present the most conservative numbers and account for possible laboratory contamination. For one brand, the three-trial average was less than the number found in the deionized blank and, thus, the final sample number was listed as zero. Brand averages ranged from 0 to 14.3 particles/L with an overall mean of 4.05 particles/L (Table 5).

**Table 5:** Summary of beer results.

	Particles Per Liter			
WATER SOURCE/ BEER ID	MINIMUM	MAXMUM	MEAN	STD. DEV.
Lake Superior 1	1	3	0.67	1.15
Lake Superior 2	1	9	4.33	4.00
Lake Superior 3	4	4	3.33	0.00
Lake Michigan 1	2	3	1.33	0.58
Lake Michigan 2	0	2	0.00	1.15
Lake Michigan 3	10	16	14.3	3.21
Lake Michigan 4	0	3	2.33	1.52
Lake Huron 1	1	2	1.33	0.58
Lake Erie 1	1	2	2.00	0.58
Lake Erie 2	3	9	3.00	3.46
Lake Ontario 1	8	9	8.00	0.58
Lake Ontario 2	4	12	8.00	4.36

**Fig 4:**
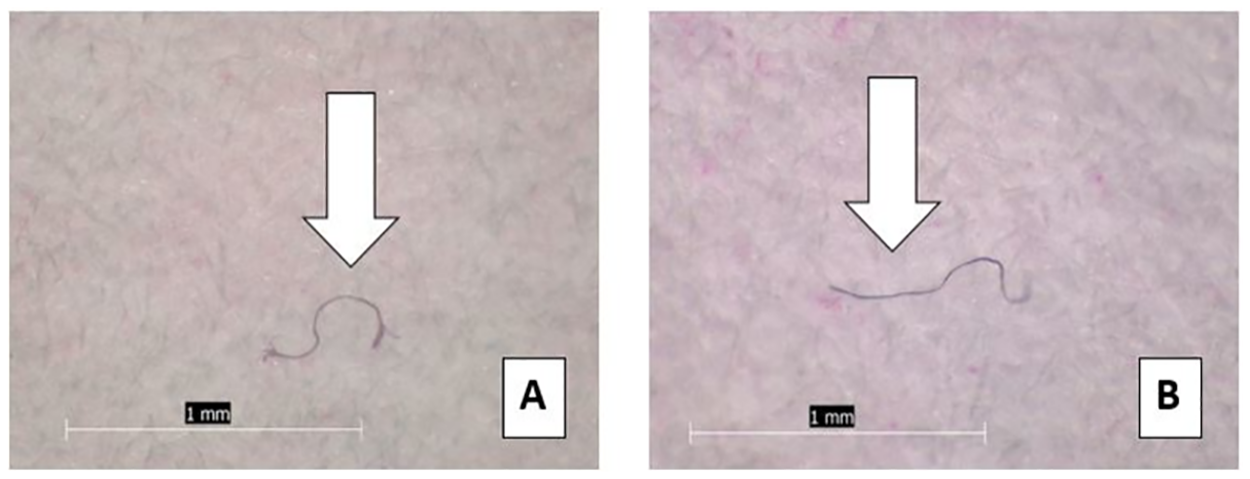
Beer particles. Examples of anthropogenic particles found in beer: (A) Fiber, 0.75 mm in length from brewery drawing water from Lake Ontario; (B) Fiber, 1 mm in length from brewery drawing water from Lake Erie.

**Fig 5:**
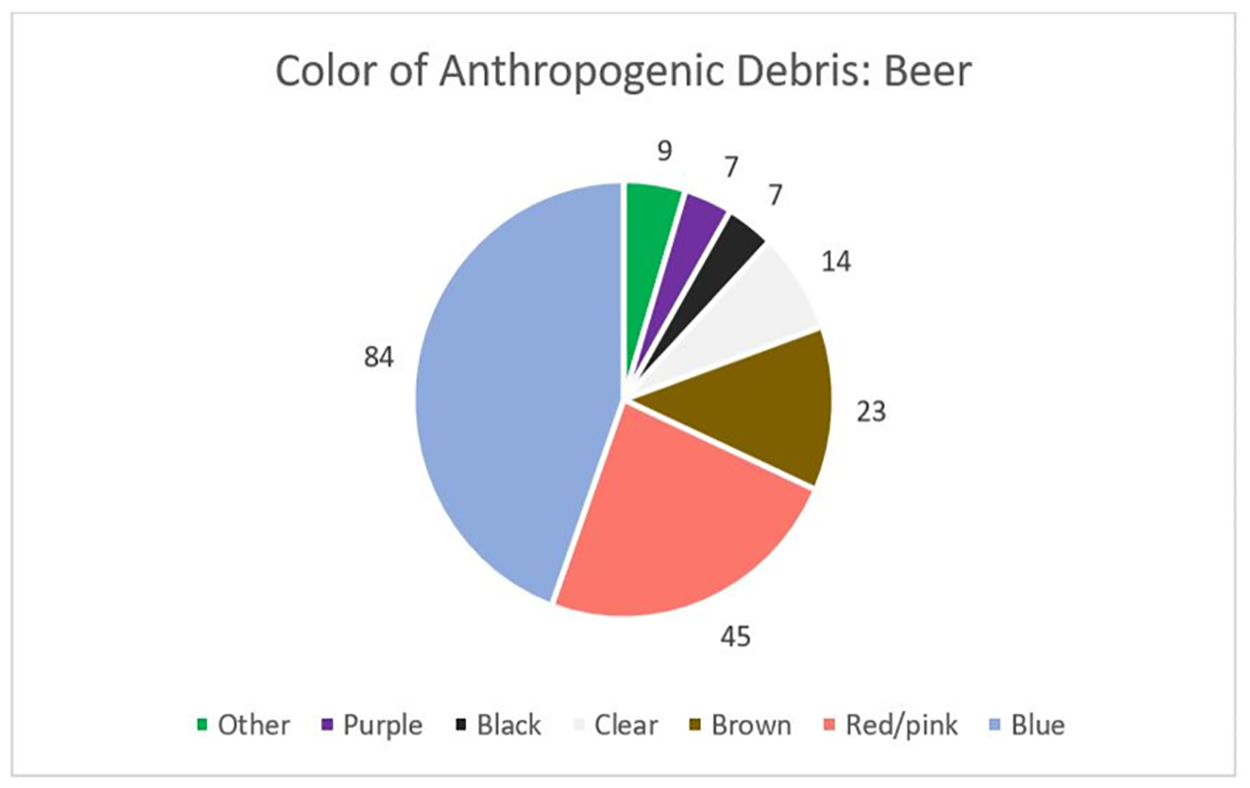
Beer particle colors. Color distribution of anthropogenic particles extracted from 12 brands of beer.

Of the 189 particles identified, the vast majority (98.4%) were classified as fibers while the remaining particles (n = 3) were identified as fragments (Fig 4). The average length of each fiber was 0.98 mm with a range from 0.1 to 5 mm. Of the 12 beer samples, 9 had one or more particle in the second filtration step for a total of 17 particles. The average length of the fibers found in the second filtration was 0.72 mm, about 0.26 mm smaller than the length of the fibers identified within the samples as a whole. Of the 189 particles, the most common color was blue, followed by red/pink, and brown, exactly in-line with that found within the tap water study (Fig 5).

As discussed in the Methods section, in seven of the nine municipalities representing the 12 beers analyzed, tap water samples were also obtained and processed in order to assess any correlation between the two (Table 6). While both the municipal tap water and the beers analyzed all contained anthropogenic particles, there seemed to be no correlation between the two (r = 0.016), which would seem to indicate that any contamination within the beer is not just from the water used to brew the beer itself.

**Table 6:** Tap v. Beer particle counts. Comparison of anthropogenic particle count in beer and its corresponding municipal tap water.

Municipality	No. Particles in Tap Water	Average No. Particles in Beer
Duluth, Minnesota	1	2.76
Milwaukee, Wisconsin	3	1.30
Chicago, Illinois	2	14.3
Holland, Michigan	2	2.30
Alpena, Michigan	1	1.30
Buffalo, New York	1	3.00
Clayton, New York	1	8.00

### Sea salt

Anthropogenic debris was found in all 12 brands of commercial sea salt that were tested. As with beer, each brand was processed three times, and one of the samples was selected at random to be filtered a second time with the particles from the second filtration added to the sample. An average of the three trials for each brand was taken, and then the number of particles found in the corresponding deionized blank was subtracted from this average in order to report the most conservative numbers. Brand averages ranged from 46.7 to 806 particles/kg, with an overall mean of 212 particles/kg (Table 7).

**Table 7:** Summary of sea salt results.

	Particles Per 50g		Particles Per Kilogram	
SALT ID	MINIMUM	MAXIMUM	MEAN	STD. DEV
North Sea Salt	0	7	66.6	3.61
Celtic Sea Salt 1	4	7	113	1.53
Celtic Sea Salt 2	4	20	187	8.19
Sicilian Sea Salt	9	13	220	2.31
Mediterranean Sea Salt 1	4	10	133	3.06
Mediterranean Sea Salt 2	3	11	133	4.16
Utah Sea Salt	4	8	113	2.08
Himalayan Rock Salt	13	37	367	12.7
Hawaiian Sea Salt	4	5	46.7	0.58
Baja Sea Salt	6	13	173	3.79
Atlantic Sea Salt	6	14	180	4.16
Pacific Sea Salt	22	51	806	15.3

**Fig 6:**
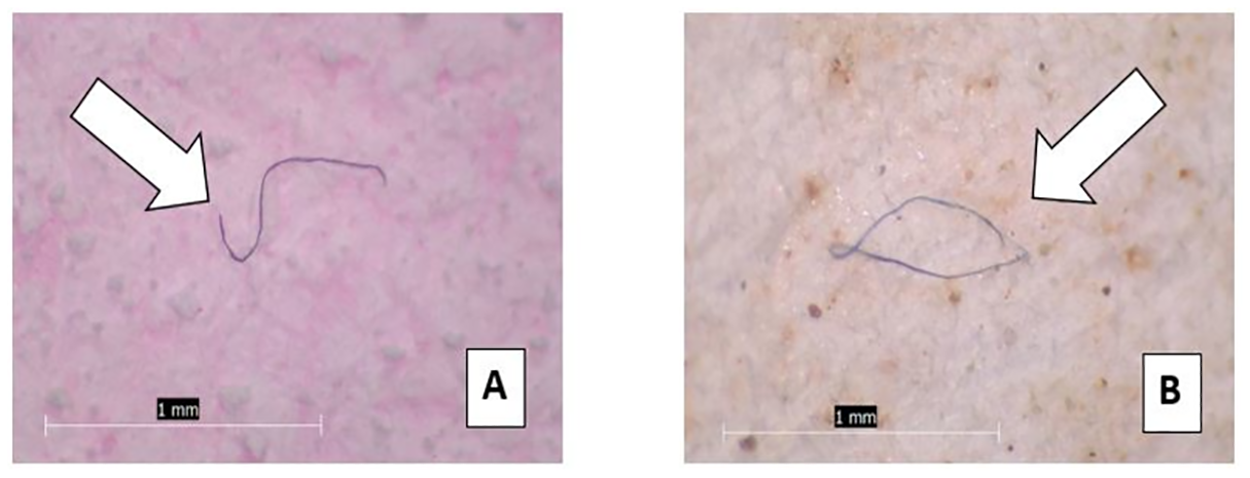
Sea salt particles. Examples of anthropogenic particles found in sea salt: (A) Fiber, 1 mm in length from Pacific Ocean sourced sea salt; (B) Fiber, 1.5 mm in length from Atlantic Ocean sourced sea salt.

**Fig 7:**
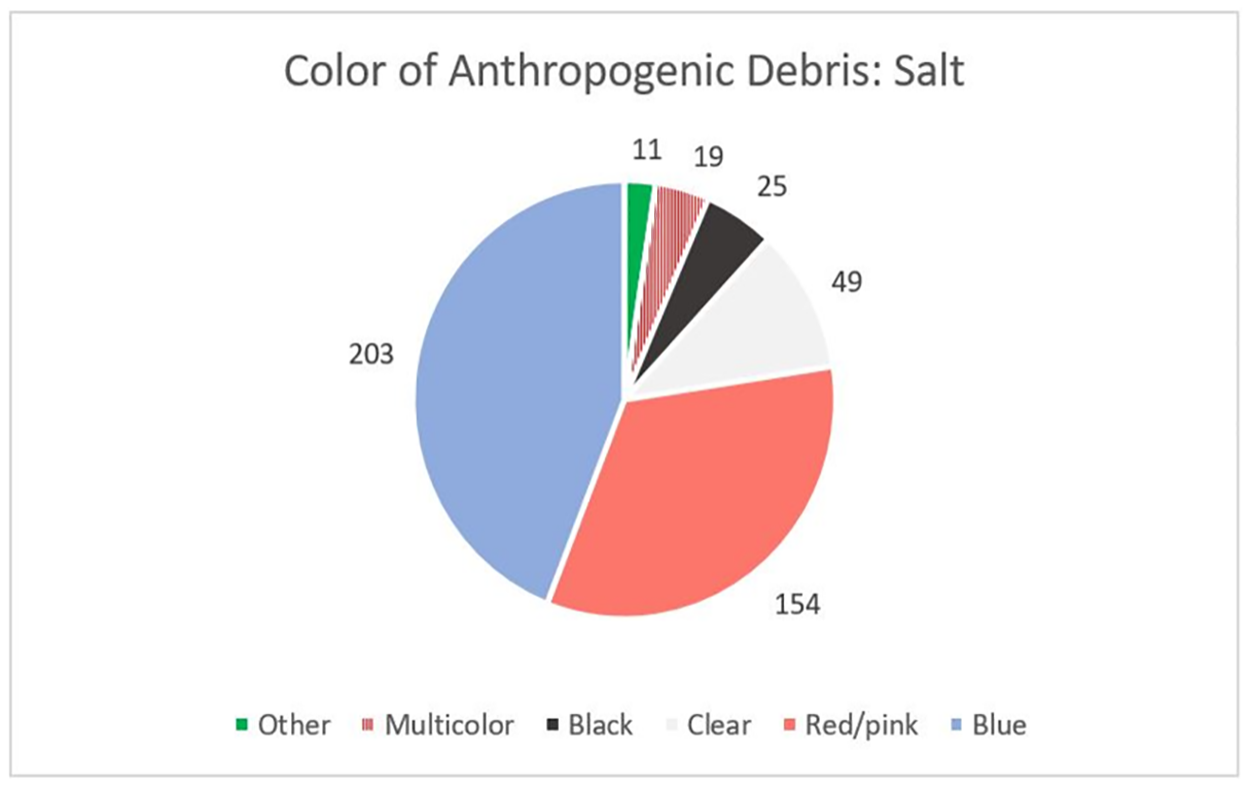
Sea salt particle colors. Color distribution of anthropogenic particles extracted from 12 brands of sea salt.

Among all samples analyzed, a total of 461 anthropogenic particles were identified. The vast majority (99.3%) of these were classified as fibers, while the remaining particles (n = 3) were identified as fragments (Fig 6). The average length of each fiber was 1.09 mm with a range of 0.1 mm to 5 mm. Five particles greater than 5 mm were omitted. Of the 12 salt samples, 8 had one or more particle in the second filtration step for a total of 23 particles. The average length of the particles found in the second filtration was 1.05 mm, about 0.04 mm smaller than the particles found in the samples as a whole. Similar to the tap water and beer results, the most common particulate color was blue, followed by red/pink, and then clear (Fig 7).

## Discussion

### Tap water

To the authors’ knowledge, this is the first survey of anthropogenic contamination of tap water to be conducted. Nearly all (81%) of the globally sourced samples for this study had anthropogenic particulate contamination far in excess of the background levels detected in the deionized blanks, 17% of which contained a single particle. When comparing anthropogenic contamination in tap water by region, North America, which includes samples from the US (n = 33) and Cuba (n = 1), had the highest mean density of anthropogenic debris at 9.18 particles/L. The lowest regional mean density of 3.60 particles/L was found in the seven EU nations of England, France, Germany, Ireland, Italy, Slovakia, and Switzerland (n = 18). Although England had the second highest mean of any country tested, it was only represented by only three samples. Of all the countries tested, the US not only had the largest sample size, it also involved samples collected from the largest geographical region (2,700 miles east to west and 1,800 miles north to south). Finally, the US dataset is unique because it includes water samples collected from municipalities that are both densely populated (8.5 million residents) and sparsely populated (5,000 residents). This stands in contrast to the samples from Ecuador, where all 24 samples were collected from the capital city.

As stated above, there was a surprising difference in the density of anthropogenic debris when comparing developed and developing nations. A higher density might be expected in developing regions that do not necessarily have municipal waste disposal and water filtration systems. However, the more developed countries together had a shared mean that was significantly higher than the developing countries. Variables such as the water source (well, surface, snowmelt), regional human population density, and water filtering methods could potentially explain this difference, but further comparative research is needed.

Although the mean density of anthropogenic debris found in bottled water was lower than the overall mean, each of the three brands was found to contain at least one particle of debris. However, the entire dataset comprised only three brands. A more expansive study of bottled water is necessary, complete with water sources and manufacturing processes.

As this is the first global survey of anthropogenic contamination of tap water, the results of this study serve as an initial glimpse rather than a comprehensive assessment. Given the ubiquity of contamination, these results are really a call for further studies within and between regions. Future studies will be designed with sampling strategies that are based on specific objectives. For example, they may focus on assessments within the water treatment process in order to better understand potential pathways of contamination. Additionally, studies could focus on different types of water sources (ground v. surface), as well as different filtration methods (reverse osmosis, mixed media, etc.), to provide insight into best practices.

According to the National Academy of Medicine, women and men should consume 2.2 L and 3 L of beverage per day, respectively. If these beverages consist of tap water, or drinks derived from tap water (such as coffee, tea, or reconstituted juice), a woman may consume as many as 12 anthropogenic particles a day, while a man could consume up to 16. These daily doses add up to an annual total of nearly 4,400 particles for women and over 5,800 particles for men. These anthropogenic particle counts are in addition to those potentially consumed in other products, such as beer, sea salt and seafood [41].

### Beer

While the tap water study represents the first of its kind, our beer study was intentionally aligned with that of Liebezeit et al. (2014) [32] but focused on Laurentian Great Lakes (USA) beers as opposed to beers from Germany. While this prior study reported 2 to 79 fibers/L of beer with an overall mean of 22.6 particles/L, our study had a narrower range, 0 to 14.3 particles/L, and a lower overall mean (4.05 particles/L). The most significant divergence between our studies, however, is that little other than fibers were found in the Great Lakes beers. The German beers, on the other hand, had 12 to 109 fragments/L and 2 to 66 granules/L in addition to the fibers. These differences may be attributable to varying brewing customs and regulations in Germany as compared to the US. In fact, a significant amount of variation in processing exists within the U.S. alone. In order to increase shelf life, national brands tend to filter their beers more thoroughly, while locally distributed craft beers may modify or forgo this step completely because they feel it affects the overall experience [42].

Even though the average number of particles found in beer (4.05 particles/L) was similar to the average number of particles found in tap water (5.45 particles/L), not even a weak correlation could be drawn when comparing the results from specific beer brands to their corresponding municipal tap water supply. In fact, the highest and lowest counts in this study came from two beers that were brewed in the same city using the same municipal water supply. This indicates that product processing may be integral to understanding anthropogenic contamination. The brand with the highest count has breweries in several states. Interestingly, the first beer samples processed in January were a different style of beer from the same company, but they were brewed in Colorado. The data for that beer were not included in our study because it did not fulfill the requirement of having its water drawn from the Great Lakes, but the mean for that beer (15.7 particles/L) was also very high. Future research efforts could focus on a particular facility, sampling at multiple locations throughout the process, in an effort to identify the source of contaminants.

It should be noted that most of the beers selected for this study were pilsners. This was intentional as wheat beers and stouts tended to clog the filters and considerably lengthen the filtration times. If there is a significant difference in the brewing process for various styles of beer, it may affect the outcome of the results and future research efforts could focus on understanding any potential differences in contamination among varieties.

In reviewing the results, it is clear that the pore size of the filter did not play a role in either the density or the size of particles detected (> 100 μm) found in the samples. Although the average length of the particle found in tap water was the smallest (0.96 mm) it was only larger by two hundredths of a millimeter when compared to the average length of particle found in beer (0.98 mm) and approximately one tenth of a millimeter when compared to the average length of particle found in salt (1.09 mm).

In order to give an indication of how many anthropogenic particles a person might consume in a year, we conducted a similar exposure analysis with beer, using averages, as we did with water. According to the average number of particles found in the 12 brands tested in this investigation, an individual consuming a single 12-fluid-ounce beer once a day could be ingesting nearly 520 particles annually. Since a slight range was found between brands, this annual ingestion could be negligible or as high as 1,800 particles.

### Sea salt

Unlike beer, there was more overlap in the ranges between the results of this investigation and the results of prior published studies on salt (Table 8). For example, Yang et al. (2015) [33] found 550 to 681 particles/kg in Chinese sea salts, while the present study found a much larger range, 46.7 to 806 particles/kg. The 2015 study also reported 7 to 204 particles/kg in Chinese rock/well salts, which was similar to the present results, 113 to 367 particles/kg, but with an overall lower count. It should be noted that only two brands of salt in this study came from inland mines, while Yang et al. (2015) [31] included five brands in this category. Slightly less than half of the particles found in the Chinese study were fibers, whereas 99.3% of the particles found in the present study were fibers.

**Table 8:** Comparison of four salt studies.

		Particles Per Kilogram			Fiber Characteristics		
REFERENCE	# BRANDS (OCEAN+SEA:MINED)	MINIMUM	MAXIMUM	MEAN	PARTICLE %	SIZE RANGE (mm)	SIZE MEAN (mm)
Yang et al. (2015)	15 (7:5)	7	681	NR^a^	majority	0.05–4.3	NR^a^
Iniguez et al. (2017)	21 (16:5)	50	280	128	majority	0.03–3.5	NR^a^
Kamari et al. (2017)	17 (17:0)	0	10	1.76	25.60%	0.16–0.98	0.52
This Study	12 (10:2)	47	806	212	99.30%	0.10–5.0	1.09

^a^NR = Not Reported

A 2017 study conducted by Iniguez et al. [35] tested 21 brands of Spanish sea salt, and similar to our findings, reported mainly fibers. This previous study also reported a range of 50 to 280 particles/kg, which is very similar to the lower range shown in our study.

Another 2017 study led by Karami et al. [34] looked at globally sourced table salt. The results from this study departed most significantly from our findings, with only 72 particles recovered from 16 brands. Of those suspected particles only 30 were identified through Raman Spectroscopy as true plastic. Another interesting difference is that only 25.6% of the polymers found in the Karami et al. study were classified as ‘filaments,’ which differs greatly from the 99.3% of fibers found in this study.

Again, in order to indicate how many anthropogenic particles a person might consume annually, we conducted an exposure analysis with salt, using averages, just as we did with beer and water. The World Health Organization recommends no more than 5,000 mg of salt daily, but the U.S. Center for Disease Control advises no more than 2,300 mg. Our calculations are based on the more conservative recommendation, since salt is something that often comes in processed and packaged food, and the salt added to manufactured foods was not represented in this study. As with beer, if the average among 12 brands is applied, an individual who purchases sea salt at a grocery store and adds it to their foods, could be ingesting an extra 180 anthropogenic particles annually. However, the present study reveals an even larger range among salt brands than beer brands, which translates into as few as 40 particles to nearly 680 particles per year.

### Overall

Based on the results of previous studies, many sizes and varieties of plastic pollution were expected. The vast majority of the anthropogenic debris found in this study, however, was fibers. Verification with FTIR has proven problematic, due to the size and dimension of the fibers. The fact that they were not stained by the Rose Bengal provides supporting evidence that the anthropogenic debris is not cellulosic and thus more likely to be synthetic/plastic, but further analysis is necessary for full confirmation.

The most abundant color detected in all three consumables tested was blue and the second most abundant color detected was pink/red. Although it was not detailed in the results above, considerable variation in tone was found in both categories. Nevertheless, it is important to recognize that this could be the result of selection bias. For example, it was difficult to detect fibers that were clear even after the cellulose filter was stained with Rose Bengal. Clear or light pink fibers could be undercounted. A significant amount of (assumed) sediment was found in some brands of commercial salts, but in the absence of FTIR or Raman Spectroscopy these particles were ignored and not included in our results.

## Conclusions

This investigation reveals troubling amounts of anthropogenic debris in global tap water, North American beer, and internationally sourced (but US purchased) sea salt. Particles were found in 81% of tap water samples, as well as in all 12 brands of beer and sea salt. These findings add to a growing body of knowledge about plastic pollution in human consumables.

As research efforts continue to explore the prevalence of synthetic polymers in human consumables, it is important that all types of plastics are studied for their ability to adsorb chemicals from the surrounding environment and/or release these and other chemical additives that are used during the manufacturing process. The majority of the research about chemical leaching and animal ingestion currently focuses on beads and fragments, and there are data gaps in the research on plastic fibers. Since this study identified such a high proportion of fibers, it is clear that future ecotoxicology tests should include increasingly prominent secondary plastics, such as fibers, especially since some of the chemicals found on and in plastics are known human toxicants.

Although generating a detailed exposure analysis was not the purpose of this study, we did extrapolate our numbers from all three products in order to provide some indication of potential exposure from a combination of three commonly consumed products, beer, salt, and tap water. Although the number of particles found independently in water, beer, and salt may not be cause for alarm, the sum of the potential exposure for all three sources combined was estimated at 5,800 particles per year. The potential ubiquity of plastic in our consumer products raises concern, especially since the highest proportion comes from drinking water (88%), followed by beer (9%), and salt (3%). The high proportion from drinking water is of particular concern because it is difficult to recommend practical strategies for avoiding ingestion. While sea salt can be reduced and beer can be avoided, drinking water is not something that can or should be eliminated or restricted, yet tap water is the most prominent source of anthropogenic debris among the three consumables analyzed in this study.
